# Cas rare de lésions cicatricielles oculaires au cours du Vogt-Koyanagi-Harada

**DOI:** 10.11604/pamj.2017.28.313.4547

**Published:** 2017-12-22

**Authors:** Adil Belmokhtar, Rajae Daoudi

**Affiliations:** 1 Université Mohamed V Souissi, Service d’Ophtalmologie A, Hôpital des Spécialités, CHU IBN Sina, Rabat, Maroc

**Keywords:** Lésions cicatricielles oculaires, Vogt-Koyanagi-Harada, affection multisystémique, Ocular scarrings, Vogt-Koyanagi-Harada, multisystem disorder

## Abstract

We here report the case of a 27-year old patient, followed-up in our Department for treatment of chronic Vogt-Koyanagi-Harada disease ( VKH disease). Fundus examination showed depigmentation of the retinal pigment epithelium and of the choroid, appearing as a pseudotumoral peripapillary lesion. Vogt-Koyanagi-Harada disease is a multisystem disorder, characterized by bilateral granulomatous panuveitis with serous exudative multifocal retinal detachment. Pathophysiology of this disease is unknown, but an immunological cellular reaction against melanocytes of the skin, the meninges, the retina, the uvea, the cochlea and the labyrinth is suspected. This disease mainly occurs in young subjects from the Far East as well as in pigmented subjects. Ocular involvement is often associated with neurological (meningeal stiffness, headache, sometimes associated with focal deficit and erebrospinal fluid (CSF) pleocytosis), auditory ( perceptive deafness) and cutaneous (vitiligo, poliosis, alopecia and canities) manifestations. It usually evolves in three phases: a prodromal phase mainly characterized by neurological signs, an acute uveitic phase, a chronic phase of convalescence characterized by choroidal and tegument depigmentation or a phase of recurrence during which subretinal neovessels and subretinal fibrosis may appear. Scarrings manifest during the chronic phase of VKH disease, which is dominated by diffuse depigmentation of the fundus of the eye, scars due to nummular chorioretinal atrophy, wheals due to diffuse depigmentation, macular scar remodeling. Pseudotumoral appearance is rare and atypical during the chronic phase of VKH disease. Treatment is based on intravenous corticosteroids followed by a cycle of oral therapy. Patient should be early treated with massive and prolonged therapy to improve prognosis.

## Image en médecine

Nous rapportons le cas d'une patiente âgée de 27 ans, suivi dans notre service pour maladie de Vogt Koyanagi Harada (maladie de VKH) au stade chronique. L'examen du fond d'œil a objectivé une dépigmentation de l'épithélium pigmentaire et de la choroïde avec un aspect pseudotumoral péri papillaire. La maladie de Vogt-Koyanagi-Harada est une affection multisystémique, caractérisée par une panuvéite granulomateuse bilatérale avec décollement séreux exsudatif multifocal. On ne sait pas la physiopathologie exacte de cette maladie, mais on suspecte une réaction immunologique cellulaire, contre les mélanocytes, qui se trouvent au niveau de la peau, les méninges, la rétine, l'uvée, la cochlée et le labyrinth. Cette affection atteint préférentiellement les sujets d'âge jeune d'Extrême-Orient ainsi que les sujets pigmentés. L'atteinte oculaire est associée souvent à des manifestations: neurologiques (raideur méningée, céphalées, parfois associés à des signes déficitaires focaux et pléocytose du LCR), auditives (surdité de perception) et cutanées (vitiligo, poliose, alopécie et canitie). Elle évolue classiquement en trois phases: une phase de prodromes où on retrouve surtout des signes neurologiques, une phase uvéitique aiguë, une phase chronique de convalescence caractérisée par une dépigmentation de la choroide et des téguments ou une phase de recurrence où on peut voir apparaitre: des néovaisseaux sous rétiniens et une fibrose sous rétinienne. Les lésions cicatricielles sont retrouvées systématiquement au cours de la phase chronique de la maladie de VKH dominées par une dépigmentation diffuse du fond d'œil, cicatrices d'atrophie choriorétinienne nummulaires, plages de dépigmentation diffuses, remaniement cicatriciel maculaire. L'aspect pseudotumoral est un aspect rare et atypique au cours de la phase chronique de la maladie de VKH. Le traitement repose sur la corticothérapie intraveineuse suivie par un relais par voie orale. Elle doit étre précoce, massive et prolongée. Un traitement précoce permet un meilleur prognostic.

**Figure 1:**
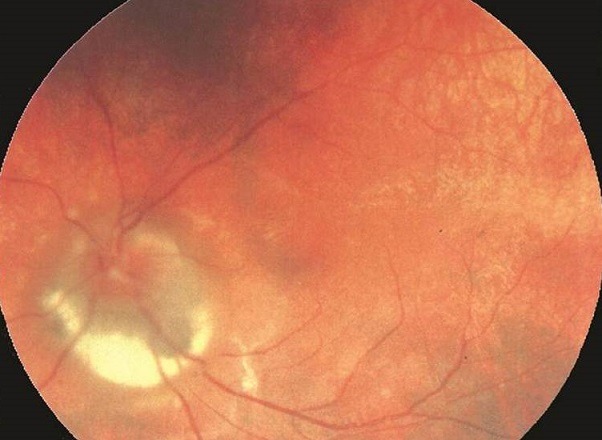
l'examen du fond d'œil d'une patiente agée de 27 ans suivie dans notre service pour maladie de VKH qui a objectivé une dépigmentation de l'épithélium pigmentaire et de la choroïde avec un aspect pseudotumoral péripapillaire (flèche)

